# Autoantibody signatures in children with celiac disease, juvenile idiopathic arthritis, and polyautoimmunity

**DOI:** 10.1002/jpr3.70119

**Published:** 2025-11-23

**Authors:** Nan Du, Denis Chang, Madison Wong, Jay R. Thiagarajah, Erin Janssen, Lauren A. Henderson, Krishnan Raghunathan, Jocelyn A. Silvester

**Affiliations:** ^1^ Division of Gastroenterology and Nutrition Boston Children's Hospital, Harvard Medical School Boston Massachusetts USA; ^2^ Department of Pediatrics, Michigan Medicine, C.S. Mott Children's Hospital University of Michigan Medical School Ann Arbor Michigan USA; ^3^ Division of Immunology Boston Children's Hospital, Harvard Medical School Boston Massachusetts USA

**Keywords:** autoimmune disorders, biomarkers, multiplex arrays

## Abstract

**Objective:**

To determine if multiplex autoantibody arrays can identify novel biomarker signatures in children with one or multiple autoimmune diseases (polyautoimmunity).

**Methods:**

Plasma collected from children (18 years or younger) in the Boston Children's Hospital Precision Link Biobank for Health Discovery between January 2007 and June 2021 was analyzed using a microarray with 120 autoantigens associated with various autoimmune diseases to assess for both immunoglobulin G (IgG) and A (IgA) autoantibodies. To determine if disease‐specific, we binary classified those with polyautoimmunity and the most common autoimmune diseases in our cohort (autoimmune thyroid disease, type 1 diabetes, or celiac disease [CeD]). For each comparison within and between groups, the autoantibody intensity was releveled to the control and a linear model was fit.

**Results:**

Plasma was analyzed from 114 children with either CeD (*n* = 31), juvenile idiopathic arthritis (*n* = 20), polyautoimmunity (*n* = 31), or no autoimmune disease (*n* = 32). Overall, children with polyautoimmunity had higher levels of specific autoantibodies relative to the other groups. Children with CeD and polyautoimmunity expressed higher levels of H/K ATPase IgA and IgG and MDA5 IgG compared to those with CeD alone.

**Conclusions:**

Use of multiplex antigen arrays in patients with and without polyautoimmunity is a valuable tool to identify known and potentially novel disease‐antigens. Further study is needed to determine the role of H/K ATPase autoantibodies in monitoring of CeD patients.

## Abstract (highlights)

**What is Known:**

- Autoantibodies are key biomarkers of autoimmune disease.
- Individuals with an autoimmune disease are at greater risk of developing another autoimmune disease.

**What is New:**

- Children with multiple autoimmune diseases produce more autoantibodies than those with only one disease.
- Multiplex arrays can help discern novel disease‐associated autoantibodies such as H/K ATPase immunoglobulin A and MDA5 immunoglobulin G, which may help identify a subgroup of patients who require additional monitoring for other autoimmune conditions.

## 1 INTRODUCTION

Autoantibodies are key biomarkers utilized in diagnosing and monitoring autoimmune conditions.^1^, ^2^ As autoantibodies often precede clinical symptoms,^3^, ^4^ they can be used to identify and monitor for overt disease in higher risk individuals, such as those with one autoimmune disease.^5^, ^6^ Celiac disease (CeD) is a chronic autoimmune disorder characterized by loss of immune tolerance to gluten,^7^ and is over‐represented among patients with type I diabetes (T1D), and autoimmune thyroid disease (ATD).^5^, ^6^ Juvenile idiopathic arthritis (JIA), an autoimmune condition which encompasses different forms of chronic arthritis of childhood, is also known to coexist with other autoimmune conditions.^8^, ^9^, ^10^ While certain autoimmune diseases are known to cluster together, such as CeD, T1D, and ATD, determining which individuals will go on to develop additional autoimmune diagnoses (i.e., polyautoimmunity) is unknown.^11^, ^12^

Testing for multiple autoantibodies is one method for screening, but can be laborious, expensive, and inefficient. Multiplex autoantibody microarrays enable testing for many autoantibodies associated with various autoimmune diseases using a small blood sample.^13^ Prior studies have shown that children who were at risk for autoimmunity have increased levels of multiple autoantibodies.^14^, ^15^ One study identified significantly higher levels of immunoglobulin G (IgG) antibodies against 14 of the 84 autoantigens tested compared to unaffected healthy donors.^14^ Most other similar studies were limited by small sample size, which can constrain the analysis needed to delineate biomarkers that distinguish those with one autoimmune disease and polyautoimmunity.

Our study explored autoantibody profiles among children with CeD, T1D, JIA or multiple autoimmune diseases (polyautoimmunity) to identify potentially novel disease‐specific autoantibodies.

## 2 METHODS

### 2.1 Ethics statement

The Boston Children's Hospital Institutional Review Board has reviewed and determined that the research study is exempt under secondary research for which consent is not required.

### 2.2 Study population

Participants (18 years or younger) in the Boston Children's Hospital Precision Link Biobank for Health Discovery with either CeD, JIA, polyautoimmunity (i.e., multiple autoimmune disorders), or no autoimmune disease (control) were included in our cross‐sectional cohort study. Patient selection was based on the autoimmune diagnoses documented by the treating clinician in the medical record. Plasma was isolated from samples collected between January 2007 and June 2021. Children on systemic steroids (i.e., prednisone) or intravenous immunoglobulin at the time of sample collection and children with a known inborn error of immunity (e.g., polyendocrinopathy, enteropathy, X‐linked syndrome (IPEX), common variable immunodeficiency) were excluded. A chart review was performed to collect pertinent clinical data including age at sample collection, age at autoimmune disease diagnosis, medical and surgical history, dietary adherence to gluten‐free diet (for those with CeD), current medications and whether another autoimmune diagnosis was made during the follow‐up period. The three autoimmune disease diagnoses that had *n* > 10 were used for the polyautoimmunity stratification studies. Sub‐groups were defined according to their autoimmune diagnoses.

#### 2.2.1 Autoantibody detection

Plasma was analyzed for both IgG and immunoglobulin A (IgA) autoantibodies using a microarray with 120 autoantigens associated with various autoimmune diseases (UT Southwestern; Table S1). Plasma antibody binding was detected by fluorochrome labeled anti‐human IgG and IgA antibodies. Each microarray plate contained internal controls which were used to normalize signal intensity across different plates. The assay used for this study was run by the University of Texas Southwestern Microarray and Immune Phenotyping Core Facility, which has been providing high‐throughput biomarker profiling services since 2001 and have previously published on their assay technology.^13^

### 2.3 Bioinformatic analysis

All analysis was carried out using custom programs written in R (2024.04.2 Build 764). Heatmaps and principal component analysis (PCA) plots were generated using the Complex HeatMap and PCA tools packages.^16^, ^17^ Difference in autoantibody levels were analyzed statistically by modifying Limma Packages initially developed for gene expression changes.^18^ For every comparison, the antibody intensity was releveled to the control and a linear model was fit. Given the linear fit, the empirical Bayesian method was used to filter the antibodies that were significantly altered between conditions. After the select autoantibodies were identified, analysis of variance (ANOVA) was used to identify the autoantibodies that were significantly different across the groups, followed by individual *t*‐tests for comparison between groups. Global method of testing a *p*‐value of 0.05 was used as a cutoff. Boxplots were generated using Graphpad Prism 10.

### 2.4 Statistical analysis

Standard descriptive statistics were used (i.e., medians, means, and standard deviations for continuous variables and proportions for categorical variables). ANOVA and unpaired *t*‐test with a *p*‐value of 0.05 was used as a cutoff to identify statistically significant autoantibodies.

## 3 RESULTS

### 3.1 Cohort characteristics

Plasma was analyzed from 114 children, including those with no autoimmune disease (*n* = 32), CeD (*n* = 31), JIA (*n* = 20), and polyautoimmunity (*n* = 31). Most children were female (73/114, 64%) and the mean age at sample collection was 11.9 ± 4.3 years.

### 3.2 Controls

The most common comorbidities among the 32 children without autoimmunity were atopic conditions (e.g., asthma, eczema, food allergies) (*n* = 8). Other co‐morbid conditions in this group are listed in Table S2.

### 3.3 CeD

The median time from CeD diagnosis to sample collection was 3.3 years (interquartile range [IQR] 1.1–5.5 years). A total of 28/31 patients (90.3%) had documented strict adherence to a gluten‐free diet at the time of sample collection. One patient who was diagnosed at the time of sample collection had not yet started on a gluten‐free diet. Two patients had documented poor compliance to a gluten‐free diet.

### 3.4 JIA

Of the 20 JIA patients, 9 (45%) had oligoarticular JIA while the other 11 (55%) had polyarticular JIA. A total of 12/20 patients (60%) had positive antinuclear antibodies at the time of their initial JIA diagnosis. Biologics (tocilizumab, adalimumab, and infliximab) were the most common treatment (*n* = 13), followed by methotrexate (*n* = 3), NSAIDs (*n* = 2), Janus kinase inhibitor (tofacitinib) (*n* = 1), and leflunomide (*n* = 1). The median time from JIA diagnosis to sample collection was 4.4 years (IQR 0.9–7.7). One patient was diagnosed with JIA approximately 5 months following sample collection as they were initially diagnosed with septic arthritis.

### 3.5 Polyautoimmunity group

Most patients in the polyautoimmune group had two autoimmune conditions (24/31, 77%) though some had three diagnoses (7/31, 23%) (Figure S1). ATD and T1D were the most common diagnoses (*n* = 14 each), followed by CeD (*n* = 13), inflammatory bowel disease (*n* = 5), autoimmune hepatitis (*n* = 5), and JIA (*n* = 4).

### 3.6 Additional autoimmune diagnoses during follow‐up

A total of 6/114 children (5.3%) developed a new autoimmune condition during the follow‐up period (median, 31 months). There were three children in the CeD group who each developed an additional autoimmune diagnosis (ATD [*n* = 1], inflammatory bowel disease [*n* = 1], and T1D [*n* = 1]). A child in the polyautoimmunity group who already had autoimmune hepatitis and JIA developed microscopic colitis. One child in the control group was diagnosed with CeD, while another developed both CeD (515 days) and ATD (504 days) following sample collection.

### 3.7 Autoantibody profiles overlap for all patients

**Figure 1:**
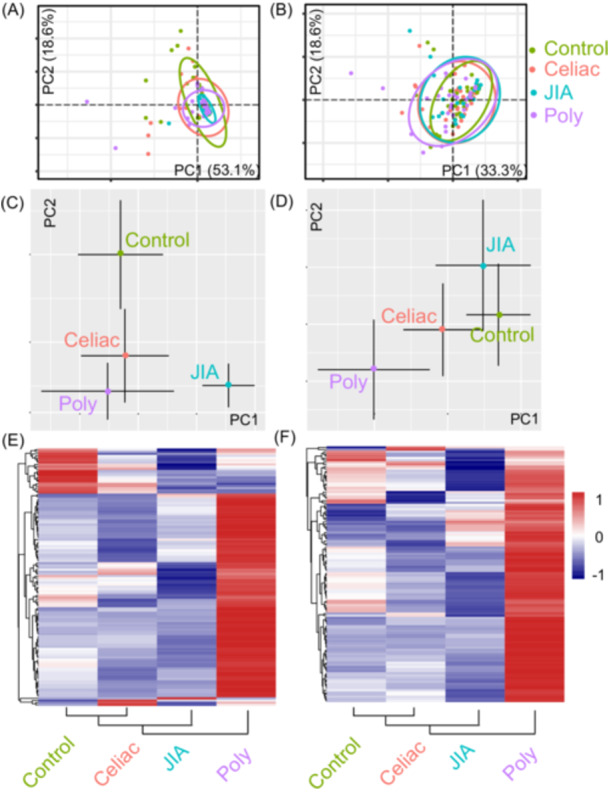
PCA for (A) IgA and (B) IgG autoantibody profiles in the four population groups (control, CeD, JIA, and polyautoimmunity). The ellipses denote the 95% confidence interval of the PCA distribution for each patient population. Median distribution of the PCAs for IgA (C) and IgG (D) antibodies shows differences between the controls and children with autoimmunity. Scaled heatmaps of the median antibody intensities for both IgA (E) and IgG (F) autoantibodies demonstrate CeD to be the closest cluster to the controls, followed by JIA, and polyautoimmunity groups. CeD, celiac disease; IgA, immunoglobulin A; IgG, immunoglobulin G; JIA, juvenile idiopathic arthritis; PCA, Principal component analysis.

A PCA was performed on the signal intensity of the microarrays. The first two principal components accounted for more than 50% of the explained variance in both IgA and IgG. The PCA plot revealed significant overlap of the four groups (CeD, JIA, polyautoimmunity, and unaffected controls; Figure 1A,B). The median distribution of the PCAs for both IgA and IgG differed between the controls and those with autoimmunity (Figure 1C,D). When the median intensity of the autoantibodies was determined for each condition and renormalized, differences between the groups were further magnified. Those in the CeD group (90.3% were on gluten‐free diet) were most like the control group while those with polyautoimmunity were most divergent as evidenced by the heatmaps and the corresponding clustering (Figure 1E,F).

Given the heterogeneous nature of the heatmap in both IgA and IgG autoantibody expression within and between groups, we reasoned that there could be distinct populations within each group that results in the PCA overlap (Figure S2). This could be either because there are affected or nonaffected populations or because there are populations that respond divergently.

### 3.8 Patients with JIA have a distinct autoantibody signature

**Figure 2:**
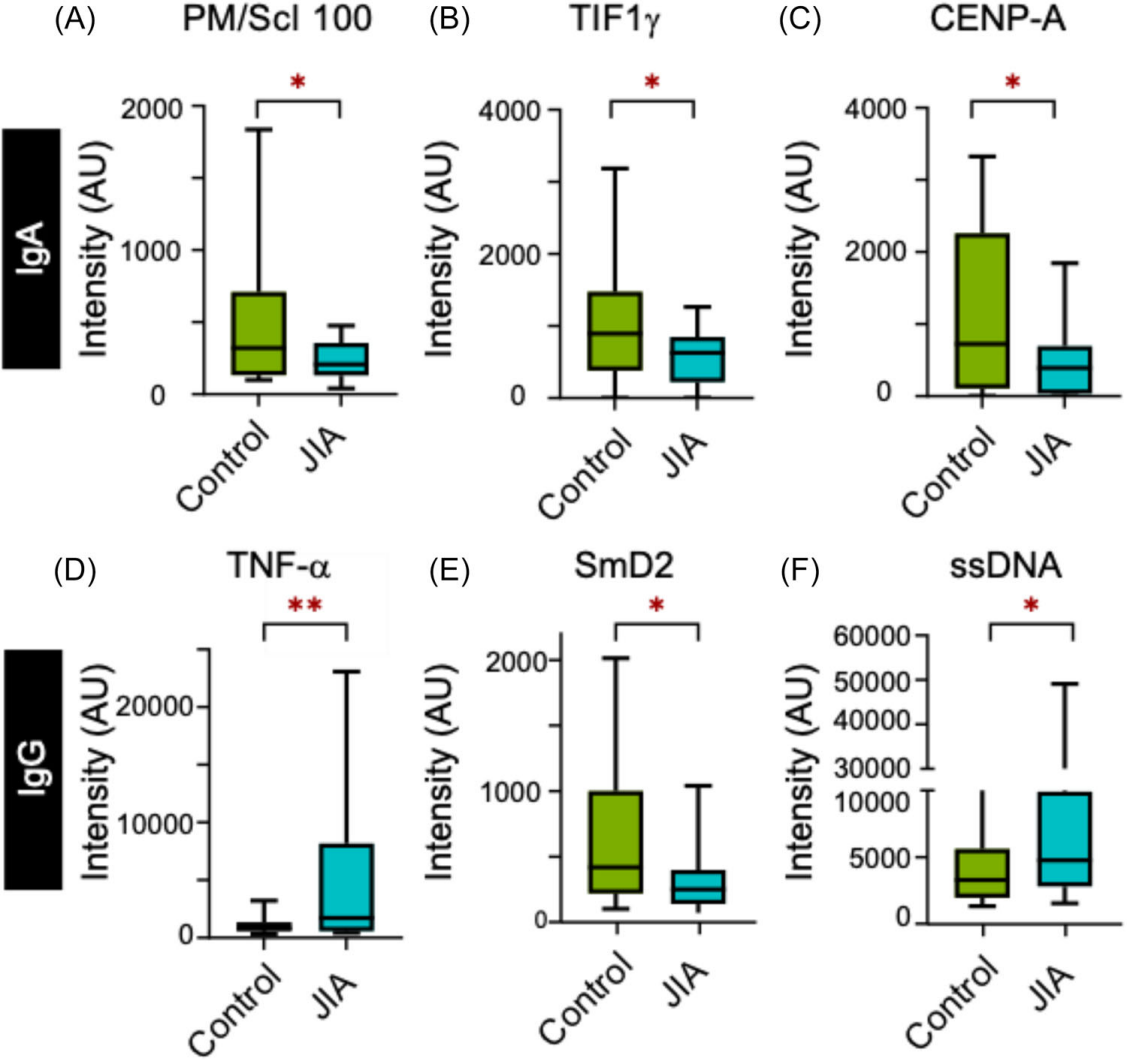
Distribution of autoantibody intensities for IgA (A–C) and IgG (D–F) autoantibodies that are significantly different between the control and JIA groups. Student *t*‐test was performed between control and JIA for each autoantibody. *p < 0.05, **p < 0.01. CeD, celiac disease; IgA, immunoglobulin A; IgG, immunoglobulin G; JIA, juvenile idiopathic arthritis.

To identify which autoantibodies were significantly distinct in the JIA group, we compared the autoantibody profiles of those with JIA to the controls. We found three IgG (tumor necrosis factor [TNF]‐α IgG, SmD2, and ssDNA) and three IgA (PM/ScL‐100, TIF1, CENP‐A) autoantibodies to be statistically different (Figure 2). On further analysis, cumulative probability plots for some autoantibodies (SmD2 IgG and TIF1 IgA) showed that the overall distribution of autoantibody intensity was shifted lower in JIA patients compared to controls (Figure S3B,E). For the other autoantibodies, the difference detected between the JIA and control group was more nuanced. Specifically, while the distributions overlapped at low intensities, the JIA and control populations diverged at higher intensities (Figure S3A,C,D, CENPA IgA, PM/Scl 100 IgA, and TNF‐α IgG).

### 3.9 Subgroup analysis of polyautoimmune patients identifies biomarkers of ATD, T1D, and CeD

We compared the polyautoimmunity group with the control group and observed that those with polyautoimmunity also had increased expression of several autoantibodies. However, unlike the JIA population, the polyautoimmunity group is an umbrella cohort with various autoimmune diseases subsumed in it. Consequently, for example, a patient with CeD would likely have a distinct profile compared to an individual with ATD. Thus, identifying a specific disease signature across these different polyautoimmunity diseases may not be necessarily complete.

### 3.10 Stratification of ATD among polyautoimmune group

**Figure 3:**
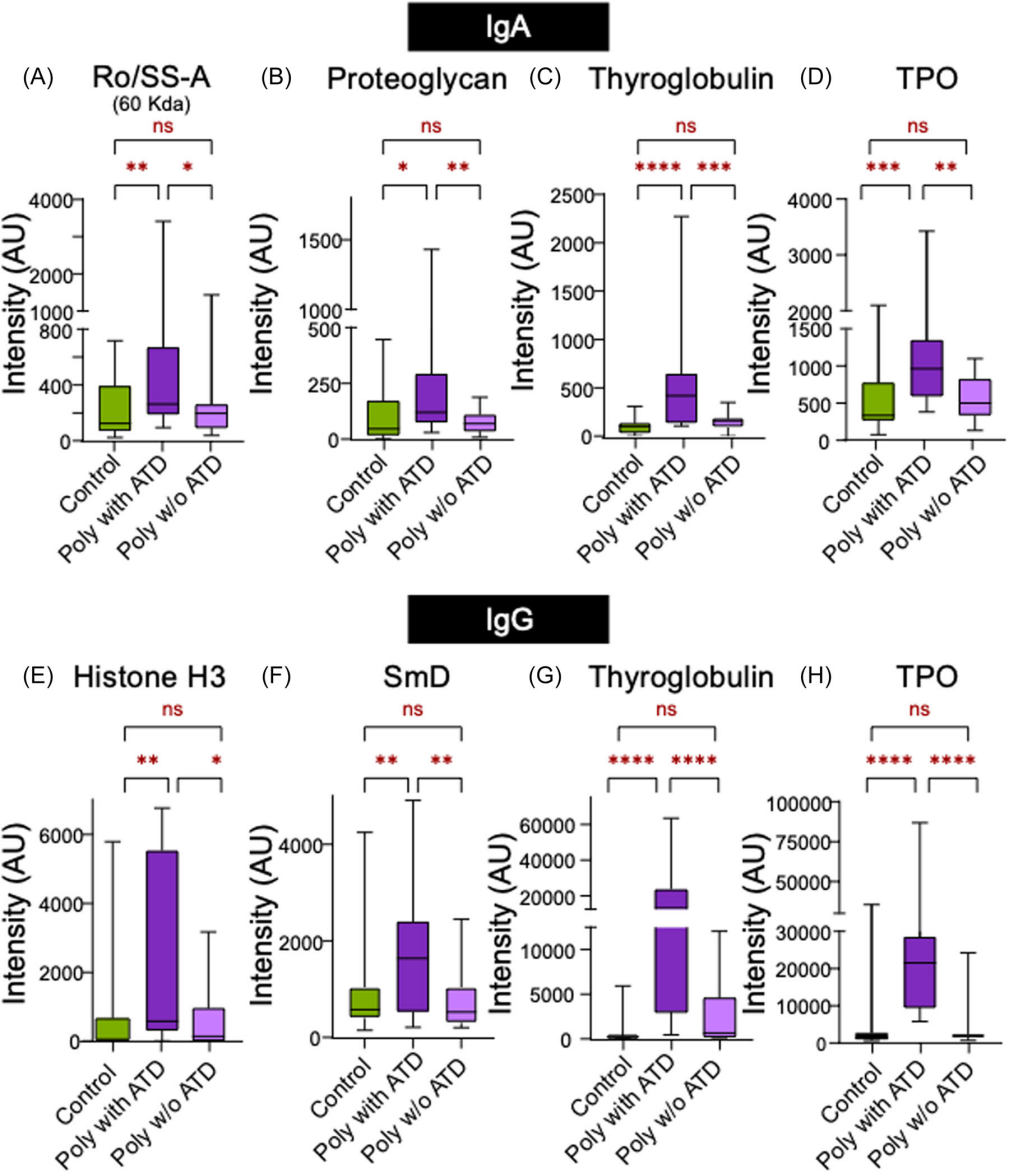
Comparison of autoantibody intensities that differ significantly among polyautoimmune children with and without ATD. (A–D) corresponds to IgA autoantibodies while (E–H) corresponds to IgG autoantibodies. First, ANOVA test was performed amongst three groups for each autoantibody: (A) Ro/SS‐A IgA (*p* = 0.022), (B) proteoglycan IgA (*p* = 0.018), (C) thyroglobulin IgA (*p* < 0.0001), (D) TPO IgA (*p* = 0.002), (E) Histone IgG (*p* = 0.014), (F) SmD IgG (*p* = 0.012), (G) thyroglobulin IgG (*p* < 0.0001), and (H) TPO IgG (*p* < 0.0001). Further individual *t*‐tests were performed between each group with *p* < 0.05 being represented as * and *p* < 0.01 as **, *p* < 0.001 as ***, and *p* < 0.0001 as **** on the figure. ANOVA, analysis of variance; ATD, autoimmune thyroid disease; IgA, immunoglobulin A; IgG, immunoglobulin G; TPO, thyroid peroxidase.

ATD, T1D, and CeD were common among our cohort with polyautoimmunity. Therefore, we delineated the autoimmune signature of polyautoimmunity patients with and without each of these diseases (Figure S4). First, the polyautoimmune group was further stratified to those with and without ATD and both subgroups were compared to the control population. We observed two types of autoantibody signatures, one which was common to polyautoimmunity and occurred in the sub‐groups with and without thyroid disease, and another which was composed of autoantibodies that were specifically enriched in the polyautoimmune sub‐population with thyroid disease. The latter signature was dominated by established autoantibodies associated with ATD (thyroid peroxidase IgG [*p* < 0.0001] and IgA [*p* < 0.01], thyroglobulin IgG [*p* < 0.0001] and IgG [*p* < 0.0001]), which supports the utility of this approach in identifying disease‐associated biomarkers. We also found other autoantibodies (Ro/SSA IgA [*p* < 0.05], proteoglycan IgA [*p* < 0.05], SmD IgG [*p* < 0.05], and Histone H3 IgG [*p* < 0.05]) which were higher in those with ATD (Figure 3).

#### 3.10.1 Stratification of T1D among polyautoimmune group

Next, we utilized a similar approach to stratify those with polyautoimmunity with and without T1D. We found higher levels of Factor P, LKM, and SP100 autoantibodies in those with polyautoimmunity with or without T1D. (Figure S5). Conventional T1D associated autoantibodies (e.g., islet cell cytoplasmic autoantibodies, glutamic acid decarboxylase autoantibodies, insulinoma associated two autoantibodies, and insulin autoantibodies) were not included on the microarray panel.

#### 3.10.2 Stratification of CeD among polyautoimmune group

**Figure 4:**
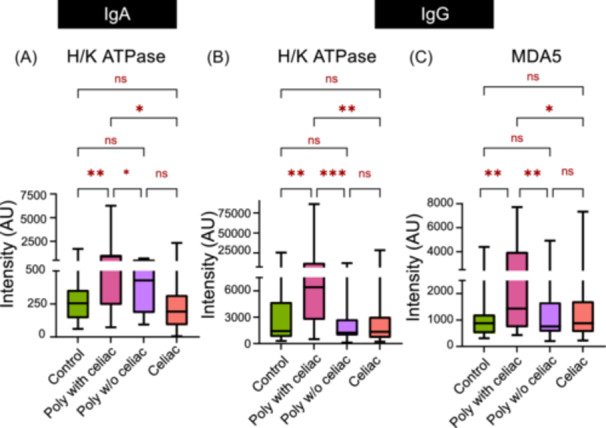
Comparison of intensity of autoantibodies that differ significantly among control, celiac disease, polyautoimmune with celiac disease and polyautoimmune without celiac disease. First, ANOVA test was performed with all groups for each autoantibody: (A) H/K ATPase IgG (*p* = 0.004), (B) H/K ATPase IgA (*p* = 0.003). (C) MDA5 IgG (*p* = 0.046). Further, individual *t*‐tests were performed between each group with *p* < 0.05 being represented as * and *p* < 0.01 as **, and *p* < 0.001 as *** on the figure. ANOVA, analysis of variance; IgA, immunoglobulin A; IgG, immunoglobulin G.

Finally, we stratified the polyautoimmune population based on the presence or absence of CeD to identify potential biomarkers of those with CeD and polyautoimmunity. For this analysis, we included those with only CeD as an additional comparison group. We were able to identify H/K ATPase and MDA5 as potentially novel autoantigens associated with CeD in those with polyautoimmunity. H/K ATPase IgA (*p* < 0.01), H/K ATPase IgG (*p* < 0.01) and MDA5 IgG (*p* < 0.05) were higher in CeD children with polyautoimmunity but not in children with CeD alone (Figure 4).

## 4 DISCUSSION

Utilizing a multiplex autoantigen microarray panel, we identified differences in autoantibody profiles in a cohort of children with one or more autoimmune conditions as well as potential novel disease associated autoantibodies. Children with CeD on a gluten‐free diet had autoantibody intensities similar to controls, which is consistent with rapid changes in the phenotype and number of gluten‐specific T cells and in circulating tissue transglutaminase (tTG) antibody levels following gluten withdrawal.^19^ Children with polyautoimmunity generally had more autoantibodies than the other groups. Thus, comparing those within the polyautoimmunity group who did or did not have a specific autoimmune disease enabled us to identify potential disease‐specific autoantibodies. Using this approach, we identified established disease‐associated autoantibodies, such as thyroid peroxidase and thyroglobulin in ATD, as well as lesser known and potentially novel autoantibodies in T1D, such as LKM1, a rare biomarker of T1DM,^20^ and SP100, a biomarker of primary biliary cirrhosis (PBC).^21^ While none of our patients with T1D also had PBC, this finding was of interest as a recent study demonstrating higher risk of PBC in individuals with T1D and vice versa.^22^

Analogously, we identified higher levels of antibodies to H/K ATPase and MDA5 in those with CeD and polyautoimmunity, but not CeD alone. H/K ATPase is an integral plasma membrane protein at the level of the intracellular secretory channels of gastric parietal cells.^23^ Autoantibodies to H/K ATPase have been described in several autoimmune disorders including autoimmune gastritis, autoimmune thyroiditis, T1D, vitiligo, and autoimmune hepatitis, which may be due to similar genetic HLA haplotypes.^24^, ^25^ However, none of our CeD patients with polyautoimmunity had any of those conditions. Interestingly, H/K ATPase antibodies are strongly associated with pernicious anemia which results in Vitamin B12, folate and iron deficiency, which are also common in untreated CeD, though the etiology is most likely due to underlying enteropathy.^26^, ^27^, ^28^, ^29^ Thus, autoantibodies to H/K ATPase may be useful in risk stratification for increased lab monitoring.

We also identified a previously highlighted association of antibodies to MDA5 and CeD.^30^ In a cross‐sectional study of adults with inflammatory myopathy, subjects with anti‐MDA5 antibodies were more likely to have positive anti‐tTG IgA antibodies, suggestive of CeD (OR 6.41). Additional research is needed to determine whether the presence of anti‐MDA5 in CeD patients confer an increased risk of developing idiopathic inflammatory myopathies in the future or if the other autoantibodies identified in our study can identify those at risk of developing polyautoimmunity.

Strengths of our study include the inclusion of a relatively large and well‐defined cohort with longitudinal clinical data. Our study population was treated at a single tertiary pediatric hospital center, which enabled us to include a large cohort of children with polyautoimmunity without known predisposing genetic mutations, but may limit generalizability. We also have a very heterogeneous polyautoimmune group, and an even larger population study would be able to more accurately classify patient subgroups and their development of autoantibodies. We also had only one blood sample for most patients, thus we were unable to assess how autoantibodies fluctuate over time in individuals (off and on treatment). Additional longitudinal studies would be helpful to determine the sequence of antibody development in polyautoimmune patients, especially given the interval between diagnosis and sample collection in our study. Inclusion of children with active, untreated CeD would have been helpful in establishing whether the antibody was present at diagnosis or emerged after commencing a gluten‐free diet. Furthermore, we did not replicate these findings with a validation cohort and did not have specific information on affinity of these autoantibodies.

## 5 CONCLUSIONS

Our study is one of the first to utilize a multiplex array to identify variations in autoantibodies in a well‐defined pediatric cohort inclusive of children with one or multiple autoimmune diseases. We demonstrate that there may be value in utilizing multiplex arrays to discern autoantibody profiles in patients with polyautoimmunity, and identified novel antigens that could be used to identify patients who may go on to develop another autoimmune disease. Further work is still needed to evaluate if H/K ATPase and anti‐MDA5 antibodies can be clinically relevant biomarkers of CeD in untreated patients or useful to screen for inflammatory myopathies in children with CeD.

## CONFLICT OF INTEREST STATEMENT

The authors declare no conflicts of interest.

## Supporting information

Supporting information.

Supporting information.

Supporting information.

Supporting information.

Supporting information.

Supporting information.

Supporting information.

Supporting information.
