# Supplementary material for: Autoantibody signatures in children with celiac disease, juvenile idiopathic arthritis, and polyautoimmunity
Source: JPGN Rep. 2025 Nov 23;7(1):118–26. doi: 10.1002/jpr3.70119 (PMC12894067; doi:10.1002/jpr3.70119)
Supplement: Supplementary file 1 — Supporting information. [file JPR3-7-118-s004.docx]

**Supplemental Table S1: List of 120 Autoantigens on the Microarray Super Panel**

| ACE2 | DFS70 | KU (P70/P80) | Proteoglycan |
| --- | --- | --- | --- |
| Aggrecan | dsDNA | La/SS-B | Prothrombin |
| Albumin | EJ | Laminin | Ro/SS-A(52 kDa) |
| Alpha Fodrin | FACTOR B | LC1 | Ro/SS-A(60 Kda) |
| Amyloid Beta(1-40) | FACTOR H | LKM 1 | SAE1/SAE2 |
| Amyloid Beta(1-42) | FACTOR I | LPS | Scl-70 |
| AQP4 | FACTOR P | Lysozyme | SLA/LP |
| BAFF | Fibrinogen Type I-S | M2 | Sm |
| BCOADC-E2 | Fibronectin | MBP | Sm/RNP |
| BPI | GAD65 | MDA5 | SmD |
| Calprotectin/S100 | GBM | Mi-2 | SmD1 |
| CD4 | Genomic DNA | Mitochondrion | SmD2 |
| CD40 | Gliadin | MPO | SmD3 |
| CENP-A | gp210 | Myosin | SP100 |
| CENP-B | GP2 | Nrp1 | SRP54 |
| Collagen I | H/K-ATPase | Nucleolin | ssDNA |
| Collagen II | Histone | Nucleosome | Tau |
| Collagen III | Histone H1 | Nup 62 | Thyroglobulin |
| Collagen IV | Histone H2A | NXP2 | TIF1 gamma |
| Collagen V | Histone H2B | OGDC-E2 | TLR4 |
| Complement C1q | Histone H3 | P0 | TNF-alpha |
| Complement C3 | HSPG | P1 | TPO |
| Complement C4 | IA-2 | P2 | tTG |
| Complement C5 | IF | PCNA | U1-snRNP 68/70kDa |
| Complement C6 | IFN-gamma | PDC-E2 | U1-snRNP A |
| Complement C7 | IL-6 | PL-7 | U1-snRNP C |
| Complement C8 | IL-12/NKSF | PL-12 | U-snRNP B/B' |
| Complement C9 | IL-17A | PM/Scl-75 | Vimentin |
| CRP | Jo-1 | PM/Scl 100 | Vitronectin |
| Cytochrome C | KS | PR3 | ß2-Glycoprotein 1 |
