# Supplementary material for: Autoantibody signatures in children with celiac disease, juvenile idiopathic arthritis, and polyautoimmunity
Source: JPGN Rep. 2025 Nov 23;7(1):118–26. doi: 10.1002/jpr3.70119 (PMC12894067; doi:10.1002/jpr3.70119)
Supplement: Supplementary file 3 — Supporting information. [file JPR3-7-118-s007.docx]

**Supplemental Table S2: Other Co-morbid Conditions**

| **Other Co-morbid conditions** |
| --- |
| Anxiety, OCD, ADHD |
| Sleep Apnea |
| Atopic Dermatitis |
| Obesity |
| Constipation |
| Pubertal Blockage |
| Idiopathic scoliosis |
| History of Poor Growth/ Low BMI |
| History of Kidney Stones |
| Abdominal Pain |
| Gender dysphoria |
| Vomiting |
| Hip Dysplasia |
| Resolved AKI |
