# Supplementary figures and images for: Autoantibody signatures in children with celiac disease, juvenile idiopathic arthritis, and polyautoimmunity
Source: JPGN Rep. 2025 Nov 23;7(1):118–26. doi: 10.1002/jpr3.70119 (PMC12894067; doi:10.1002/jpr3.70119)

(A)

IgA

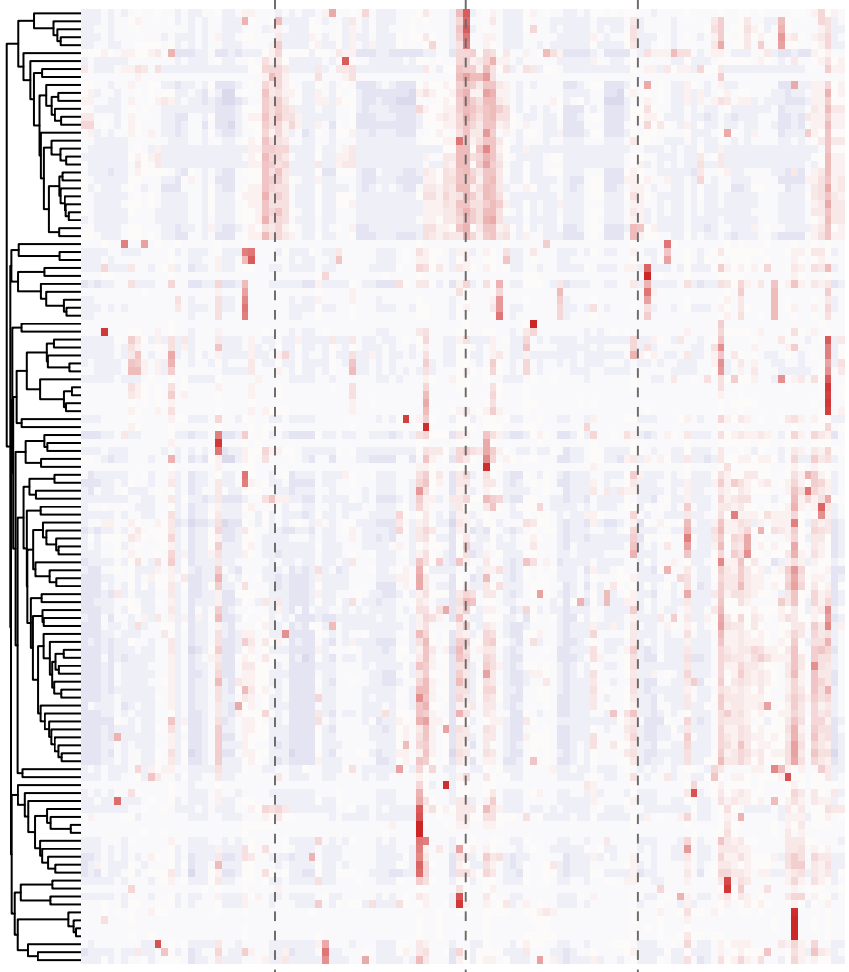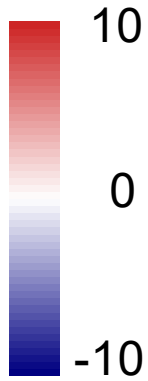

(B)

IgG

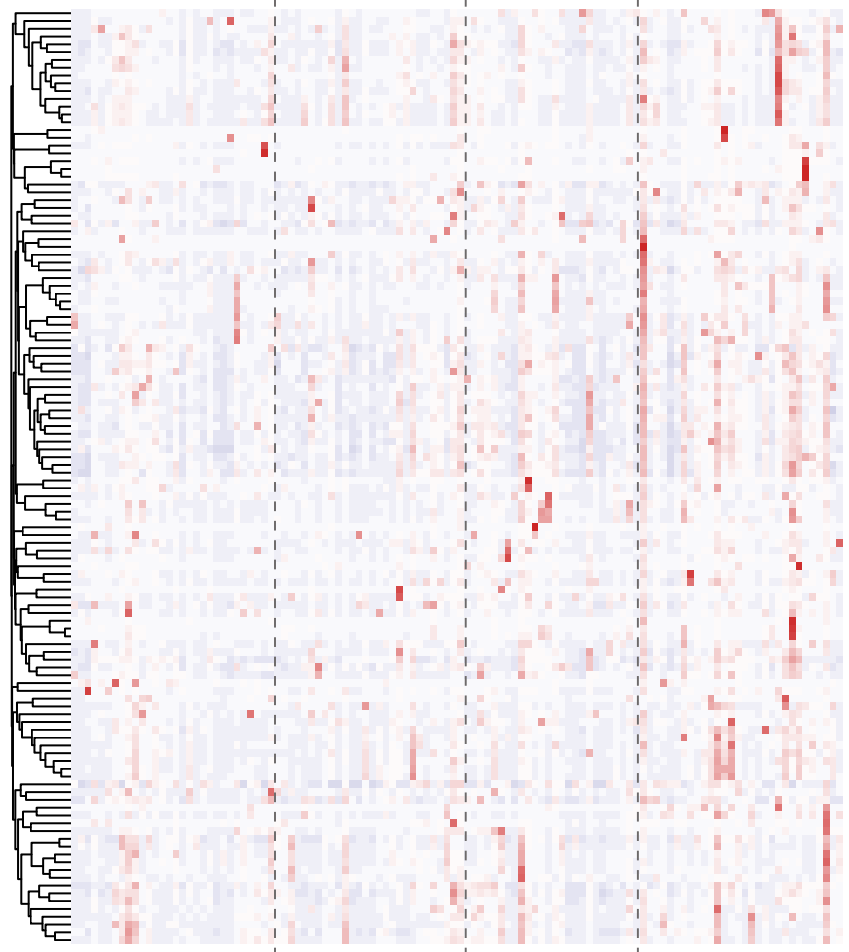

Celiac

Control

JIA

Poly

Supplement: Supplementary file 4 — Supporting information. [file JPR3-7-118-s003.pdf]

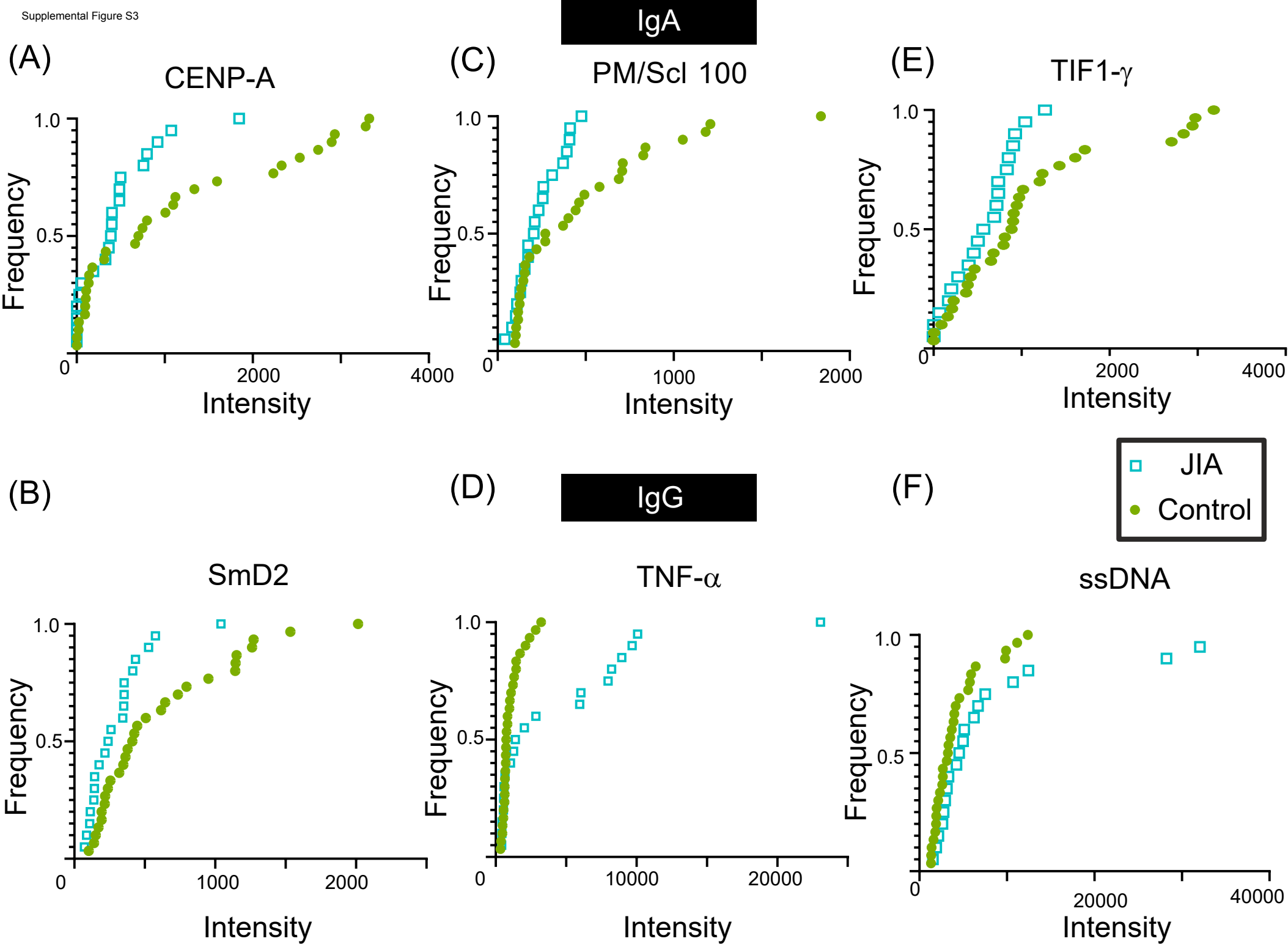

Supplement: Supplementary file 5 — Supporting information. [file JPR3-7-118-s002.pdf]

(A) Collagen IV

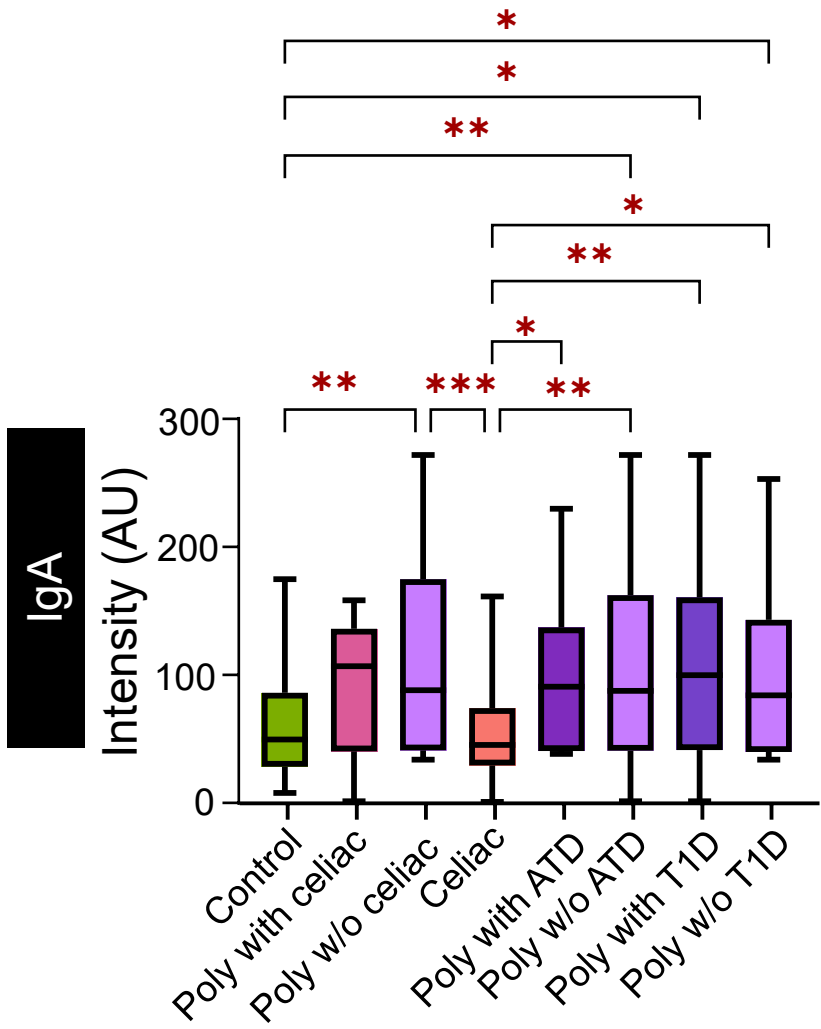

(B) Collagen I

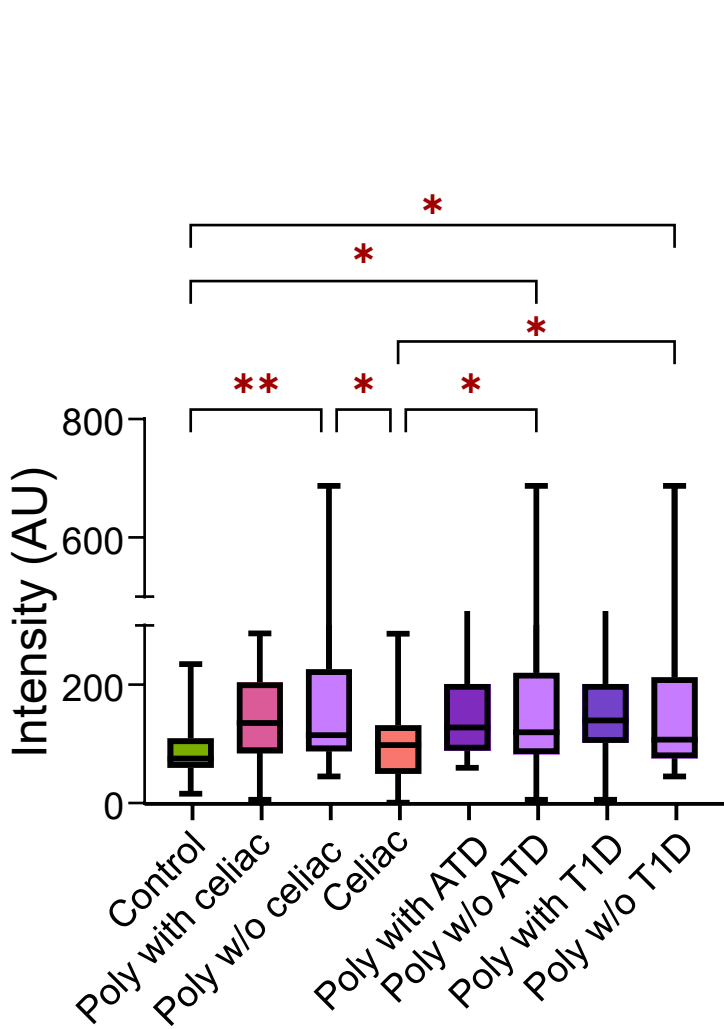

(C) TIF1 $\gamma$

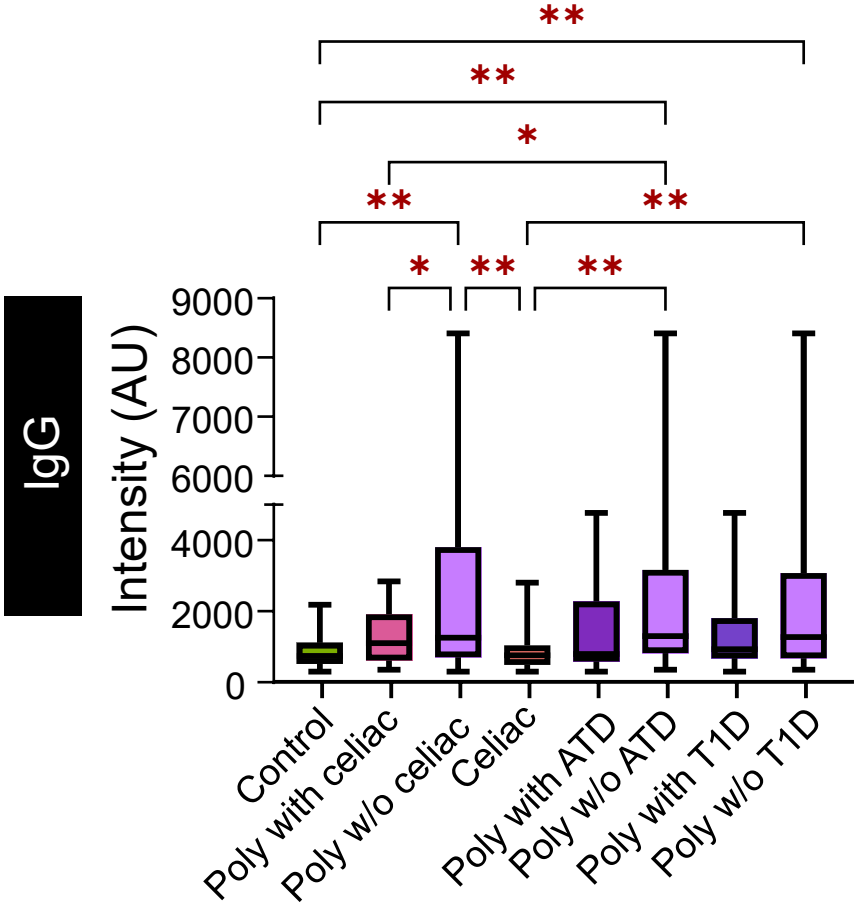

(D) TNF- $\alpha$

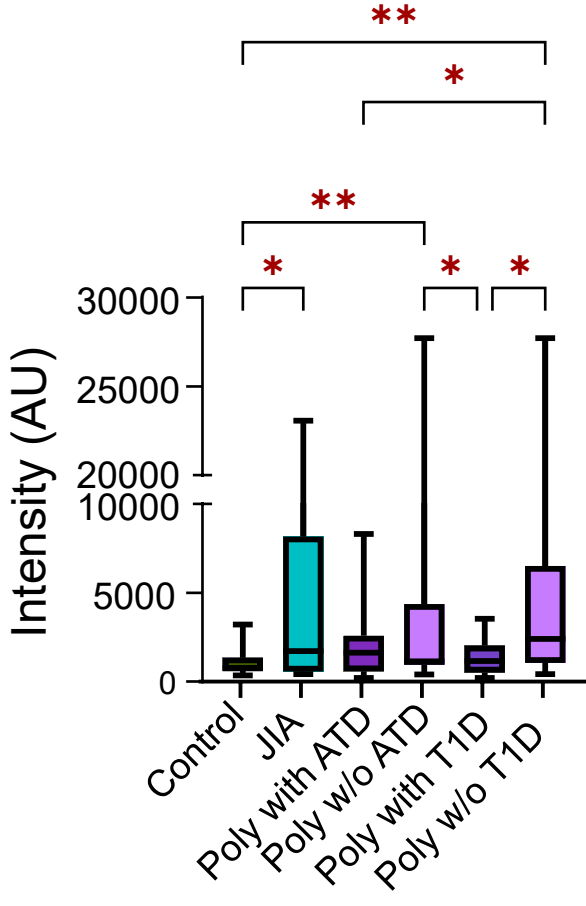

(E) U-snRNP B/B'

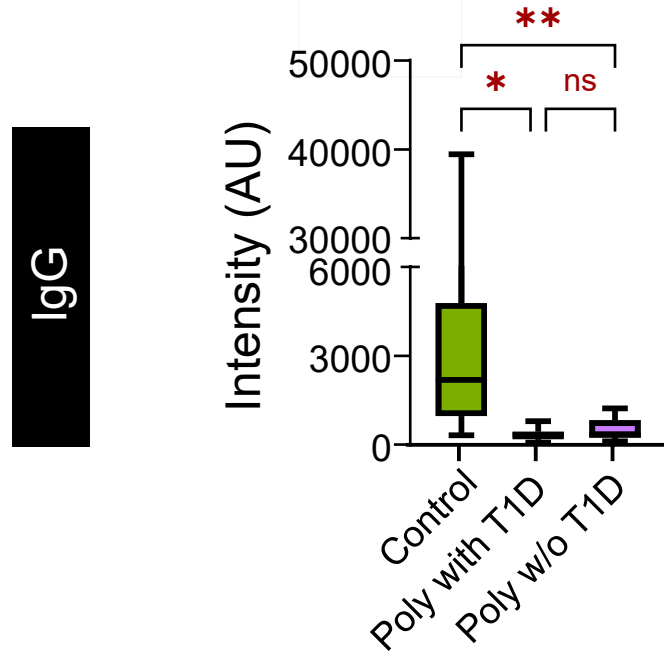

Supplement: Supplementary file 6 — Supporting information. [file JPR3-7-118-s001.pdf]

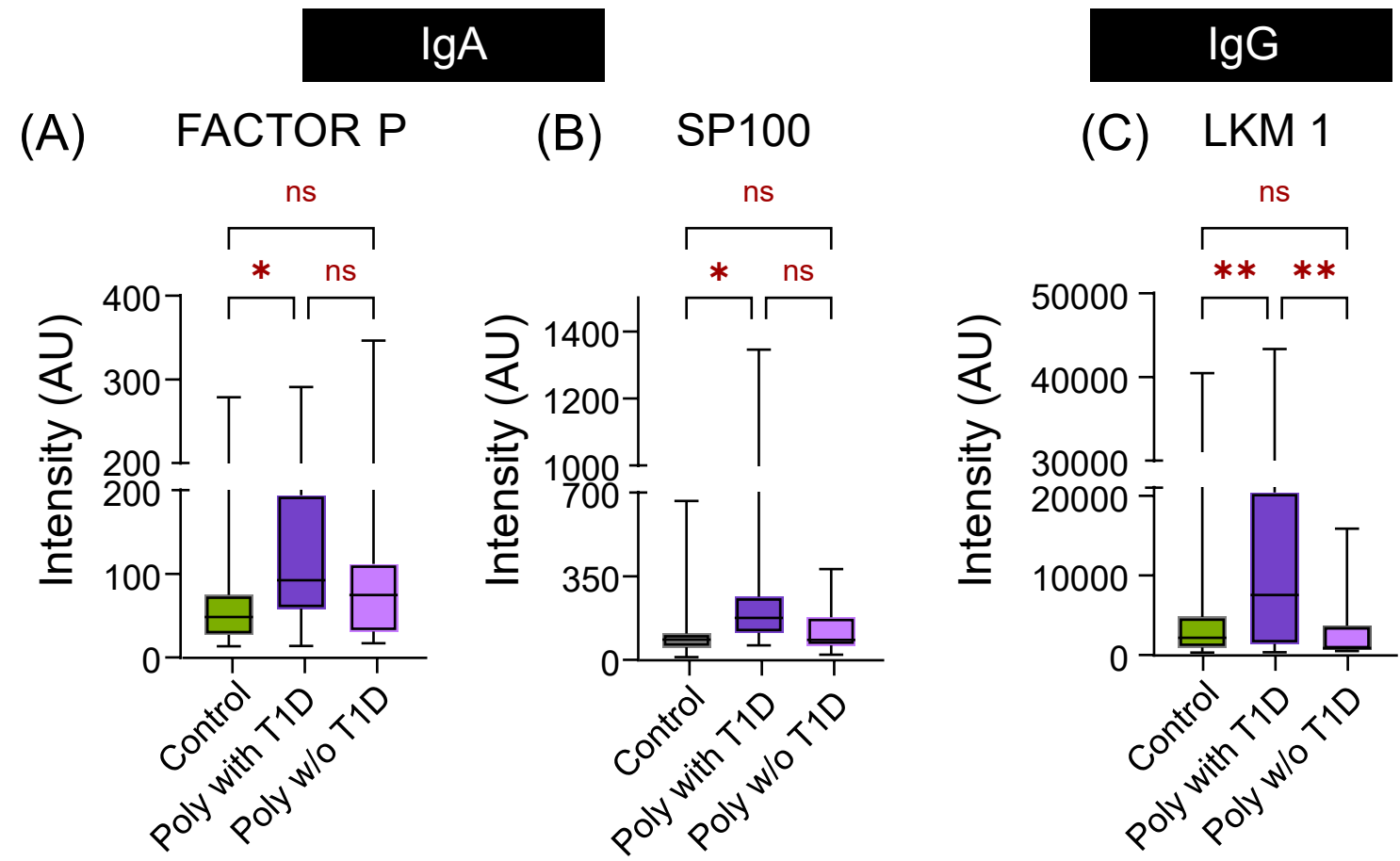

Supplement: Supplementary file 7 — Supporting information. [file JPR3-7-118-s005.pdf]
