# Supplementary material for: Autoantibody signatures in children with celiac disease, juvenile idiopathic arthritis, and polyautoimmunity
Source: JPGN Rep. 2025 Nov 23;7(1):118–26. doi: 10.1002/jpr3.70119 (PMC12894067; doi:10.1002/jpr3.70119)
Supplement: Supplementary file 8 — Supporting information. [file JPR3-7-118-s006.docx]

**Supplemental Table Legends**

Supplemental Table S1: List of 120 Autoantigens on the Microarray Super Panel

Supplemental Table S2: Other Co-morbid Conditions

**Supplemental Figure Legends**

**Supplemental Figure S1:** **Distribution of autoimmune conditions among children with polyautoimmunity**

**Supplemental Figure S2**: **Autoantibody heterogeneity among all four groups for both** Immunoglobulin A(IgA) and Immunoglobulin G(IgG) **autoantibody expression.** Scaled heatmaps for (A) IgA and (B) IgG.

**Supplemental Figure S3**: **Distribution of IgA and IgG autoantibody intensities in control and JIA patients**. The frequency distribution diverges at higher intensities for CENP-A IgA(A), PM/Scl 100 IgA(C), and TNF-alpha IgG(D). In contrast, TIF1-y IgA(E), SmD2 IgG(B) and ssDNA IgG(F) diverge at lower intensities.

**Supplemental Figure S4**: Comparison of autoantibodies that are altered between control and polyautoimmune stratified by their different comorbidities. (A)-(B) corresponds to be IgA while (C)-(D) corresponds to IgG. First, analysis of variance(ANOVA) was used to compare amongst the groups for each autoantibody: (A) Collagen IV IgA (p value= 0.001), (B) Collagen I IgA (p value=0.032), (C) TIF1y IgG (p value=0.001), (D) TNF-alpha IgG (p value = 0.010), and (E) U-snRNP B/B^’^ IgG (p value=0.008. Then further individual t-tests were performed between each group with p<0.05 being represented as * and p < 0.01 as* *, p < 0.001 as ***, and p <0.0001 as **** on the figure.

**Supplemental Figure S5**: **Comparison of autoantibody intensities that differ significantly among polyautoimmune children with and without Type 1 Diabetes (T1D).** (A) and (B) corresponds to IgA while (C) corresponds to IgG. First, ANOVA test was used to compare amongst the groups for each autoantibody: **(A)** Factor P IgA (p= 0.038), **(B)** SP 100 IgA (p=0.025) and **(C)** LKM 1 IgG (p=0.004). Control is defined as children with no autoimmune disorders. Student t-tests were performed between each group with p<0.05 being represented as * and p < 0.01 as * *, p < 0.001 as ***, and p <0.0001 as **** on the figure.
